# Posaconazole-Induced Hypertension Masquerading as Congenital Adrenal Hyperplasia in a Child with Cystic Fibrosis

**DOI:** 10.1155/2020/8153012

**Published:** 2020-08-28

**Authors:** Neha Agarwal, Louise Apperley, Norman F. Taylor, David R. Taylor, Lea Ghataore, Ellen Rumsby, Catherine Treslove, Richard Holt, Rebecca Thursfield, Senthil Senniappan

**Affiliations:** ^1^Department of Paediatric Endocrinology, Alder Hey Children's NHS Foundation Trust, Liverpool, UK; ^2^Department of Clinical Biochemistry (Viapath), King's College Hospital NHS Foundation Trust, London, UK; ^3^Department of Biochemistry, Alder Hey Children's NHS Foundation Trust, Liverpool, UK; ^4^Department of Paediatric Nephrology, Alder Hey Children's NHS Foundation Trust, Liverpool, UK; ^5^Department of Paediatric Respiratory Medicine, Alder Hey Children's NHS Foundation Trust, Liverpool, UK

## Abstract

**Background:**

Deficiency of 11*β*-hydroxylase is the second most common cause of congenital adrenal hyperplasia (CAH), presenting with hypertension, hypokalaemia, precocious puberty, and adrenal insufficiency. We report the case of a 6-year-old boy with cystic fibrosis (CF) found to have hypertension and cortisol insufficiency, which were initially suspected to be due to CAH, but were subsequently identified as being secondary to posaconazole therapy. *Case Presentation*. A 6-year-old boy with CF was noted to have developed hypertension after administration of two doses of Orkambi™ (ivacaftor/lumacaftor), which was subsequently discontinued, but the hypertension persisted. Further investigations, including echocardiogram, abdominal Doppler, thyroid function, and urinary catecholamine levels, were normal. A urine steroid profile analysis raised the possibility of CAH due to 11*β*-hydroxylase deficiency, and a standard short synacthen test (SST) revealed suboptimal cortisol response. Clinically, there were no features of androgen excess. Detailed evaluation of the medical history revealed exposure to posaconazole for more than 2 months, and the hypertension had been noted to develop two weeks after the initiation of posaconazole. Hence, posaconazole was discontinued, following which the blood pressure, cortisol response to the SST, and urine steroid profile were normalized.

**Conclusion:**

Posaconazole can induce a clinical and biochemical picture similar to CAH due to 11*β*-hydroxylase deficiency, which is reversible. It is prudent to monitor patients on posaconazole for cortisol insufficiency, hypertension, and electrolyte abnormalities.

## 1. Introduction

Congenital adrenal hyperplasia (CAH) is a group of autosomal recessive monogenic disorders, characterized by a block in the cortisol steroidogenesis pathway [1]. More than 95% of CAH cases are due to 21-hydroxylase deficiency [1]. Deficiency of 11*β*-hydroxylase is the second most common cause of CAH, accounting for approximately 5% of all cases [2]. 11*β*-hydroxylase deficiency presents with hypertension, hypokalaemia, precocious puberty, and adrenal insufficiency [2]. The cortisol deficiency leads to elevated ACTH, hyperpigmentation, and adrenal hyperplasia [1]. Features of mineralocorticoid excess (hypertension and hypokalaemia) arise due to elevated 11-deoxycorticosterone (DOC), which is generally regarded as having 1/40th the mineralocorticoid potency of aldosterone [2].

Cystic fibrosis (CF) is the most common genetic disorder in the Caucasian population (1 : 2500) [3]. CF is a multisystem, autosomal recessive disorder, caused by mutations in the cystic fibrosis transmembrane conductance regulator (CFTR) gene that encodes a chloride-conducting transmembrane channel [4]. The CFTR protein is an ion channel, promoting chloride secretion and inhibiting sodium absorption [4]. The presence of 2 mutations in CFTR is known to result in low blood pressure due to sodium loss, primarily from the sweat glands. The defective CFTR protein results in impaired mucociliary clearance and thickened bronchial secretions, leading to recurrent respiratory and gastrointestinal tract infections [5]. This impairment in mucociliary clearance and defective innate defence can lead to accumulation of fungal spores in the small airways following exposure to *Aspergillus fumigatus* [6]. This can trigger an IgE-mediated hypersensitivity response to the fungal spores, causing allergic bronchopulmonary aspergillosis (ABPA) and leading to a reduction in lung function [6].

Systemic corticosteroids are the mainstay of treatment of ABPA [6]. Some studies recommend the use of the azole group of antifungals in addition to steroids [7]. The variable pharmacokinetics of azoles in patients with CF may result in subtherapeutic or supratherapeutic drug levels in the plasma, leading to either a decreased response or an increased risk of drug-related adverse effects, respectively [8]. The treatment duration for azoles is usually prolonged, extending from weeks to months [8].

The long-term use of antifungals is known to be associated with hepatotoxicity, peripheral neuropathies, pancreatitis, and hormone-related effects [8]. Recent studies have suggested posaconazole, which is less toxic and better tolerated than other azoles, to be more effective in treating invasive fungal infection in patients with CF [7]. There are some recent reports of posaconazole-induced hypertension in adolescent and adult patients, attributed either to 11*β*-hydroxylase inhibition [9] or 11*β*-hydroxysteroid dehydrogenase 2 inhibition [10] or both [11, 12]. There are no similar reports in young children.

We report a 6-year-old boy with CF and hypertension who was initially suspected to have CAH due to 11*β*-hydroxylase deficiency but it was later identified as being secondary to posaconazole treatment. To the best of our knowledge, this is the first published report of posaconazole-induced hypertension in a young child with CF.

## 2. Case Report

A 6-year-old boy with CF was admitted for the administration of Orkambi™ (ivacaftor/lumacaftor), which acts as a potentiator of the CFTR protein. Prior to Orkambi™, elevated blood pressure (BP) readings were noted on a couple of occasions, but these were not persistent. Following Orkambi™ administration, BP was consistently high (systolic blood pressure of 150 mmHg and diastolic blood pressure of 84 mmHg, above the 99th centile for his age and height). Hypertension was initially considered to be secondary to Orkambi™ administration, which was therefore discontinued.

General history and physical examination were otherwise noncontributory, with no features pointing towards an underlying cause for high blood pressure. Plasma creatinine was normal at 40 *μ*mol/l (age-adjusted reference range: 20–57 *μ*mol/l), and plasma concentrations of sodium, potassium, and bicarbonate were also normal (140 mmol/l, 4.3 mmol/l, and 21 mmol/l, respectively). Further investigation of the cause of hypertension included an echocardiogram, abdominal ultrasound with Doppler analysis, and thyroid function studies, all of which showed no abnormality. Plasma renin (2.0 mU/L, supine reference range: 15.8 to 100.8 mU/L) and aldosterone (45.0 pmol/L, supine reference range: 80 to 970 pmol/L) levels were both low. Urinary catecholamines were not suggestive of pheochromocytoma (VMA/creatinine, 0.7 *μ*M/mM Cr (3.7–4.4); HVA/creatinine, 8–8.1 *μ*M/mM Cr (0–10); dopamine, 0.4 *μ*M/mM Cr (0–0.8); and noradrenaline, 0.03 *μ*M/mM Cr (0–0.14)).

Two weeks after discontinuation of Orkambi™, he continued to be hypertensive and was started on treatment with amlodipine (2.5 mg once daily), which maintained his blood pressure within the normal range (50th centile for his age and height).

A urinary steroid profile was sent to rule out the disorders of steroidogenesis associated with hypertension. This revealed an increased level of the 11-deoxycortisol metabolite, tetrahydro-11-deoxycortisol (261 *μ*g/mmol creatinine), and the 11-deoxycorticosterone metabolite, tetrahydro-11-deoxycorticosterone (32 *μ*g/mmol creatinine). This raised the possibility of CAH secondary to 11*β*-hydroxylase deficiency. Cortisol metabolites were present, and there was no relative increase in the androgen metabolites (DHEA < 0.4*μ*mol/L, androstenedione < 0.4 nmol/L, and testosterone < 0.7 nmol/L), which would be consistent with a mild form of such a defect.

ACTH was not elevated (3 pmol/L). A standard short synacthen test (SST) using 250 *μ*g tetracosactide demonstrated (Table 1) a suboptimal cortisol response (baseline: 269 nmol/L, 30 min: 387 nmol/L, and 60 min: 420 nmol/L) and a marked 11-deoxycortisol response (baseline: 6 nmol/L, 30 min: 102 nmol/L, and 60 min: 98 nmol/L). The peak 17-hydroxyprogesterone was normal (5 nmol/L).

Clinically, his weight was 19.6 kg (−0.31 SDS) and height was 111.6 cm (−0.80 SDS), and he was prepubertal with no evidence of pubic or axillary hair. On further evaluation of his medical history, he was noted to have been on posaconazole treatment (300 mg once/day) for nearly two and a half months. Hypertension had been documented for the first time approximately 2 weeks after the initiation of posaconazole.

To explore the possibility of posaconazole leading to the hypertension, it was discontinued for a period of 3 weeks and amlodipine was also stopped. Within a few days, the blood pressure had normalized completely. A repeat SST performed 3 weeks after the discontinuation of posaconazole revealed an adequate peak cortisol response (735 nmol/l) and normal 11-deoxycortisol concentrations (0 min: 0.6 nmol/L, 30 min: 5 nmol/L, and 60 min: 4 nmol/L). The repeat urine steroid profile was normal. Clinical and laboratory parameters on and off posaconazole treatment are summarized in Table 1.

## 3. Discussion

This case highlights the role of posaconazole in inducing a clinical and biochemical picture resembling the 11*β*-hydroxylase deficiency form of CAH. Barton et al. have reported the development of hypertensive urgency in a 15-year-old boy on prophylactic posaconazole for a combined immunodeficiency and also interpreted this as due to inhibition of 11*β*-hydroxylase activity [9]. 11*β*-hydroxylase is a mitochondrial enzyme encoded by *CYP11B1* [13]. It converts 11-deoxycortisol and 11-deoxycorticosterone to cortisol and corticosterone, respectively, by the addition of a hydroxyl group. An elevated level of 11-deoxycortisol is a robust marker to diagnose 11*β*-hydroxylase deficiency, which is characterized by inactivating mutations [13].

Convincing biochemical evidence of reversible 11*β*-hydroxylase inhibition by posaconazole was seen in our patient. Markedly increased levels of 11-deoxycortisol and 11-deoxycorticosterone metabolites in the urine steroid profile normalized 3 weeks after stopping the posaconazole treatment. This is similar to the findings in a previous study coauthored by three coauthors of this report (DT, NT, and LG, [11]) and those of Barton et al. [9]. Features that resemble CAH are bilateral adrenocortical hyperplasia, noted in all experimental animals studied [14], an increased plasma ACTH level in a 67-year-old patient on posaconazole for chronic cavitary aspergillosis of the left lung [10] and in a 67-year-old man with myelodysplastic syndrome on posaconazole prophylaxis [11], a suboptimal cortisol response to synacthen testing (this report and [11]), and a normal dexamethasone suppression test response [11]. Our patient had a normal plasma ACTH level: a comparatively shorter exposure to posaconazole than in the previous studies may explain this.

Differentiation from genetic CAH is given by finding no genetic mutation on whole genome sequencing [14], only modest increases of serum androstenedione [9, 11], or low or no change [12] with lack of clinical features of androgen excess (this report), and reversal of the biochemical changes on stopping treatment.

All studies have shown suppression of renin and aldosterone in the context of hypokalaemia, which normalized on stopping treatment. Our patient did not show hypokalaemia, but given that it is not always present in primary hyperaldosteronism, this can be accommodated [15]. The hypertension has also been successfully countered by the mineralocorticoid receptor antagonist spironolactone [11]. Mineralocorticoid excess induced by posaconazole is thus unquestionably the cause of the hypertension.

These phenomena have otherwise been interpreted as arising from apparent mineralocorticoid excess (AME) due to 11*β*-hydroxysteroid dehydrogenase 2 (11*β*-HSD2) inhibition [10, 16]. Posaconazole has been identified as a potent inhibitor in cell lysates during a virtual screening for drugs inhibiting 11*β*-HSD2 [17]. The enzyme 11*β*-HSD2 is predominantly expressed in aldosterone-sensitive tissues and converts cortisol to its inactive metabolite, cortisone [18]. Even mild defects in 11*β*-HSD2 have been shown to contribute to hypertension due to an increased intrarenal level of cortisol, which activates the type 1 mineralocorticoid receptor [18]. An elevated ratio of urinary tetrahydrocortisol (THF) and allo-tetrahydrocortisol (allo-THF) to tetrahydrocortisone (THE) is diagnostic of homozygous inactivation of the enzyme, but this change is less sensitive than an elevated serum cortisol over cortisone ratio to identify heterozygous mutations [18]. This ratio was increased in 4 posaconazole-treated patients [10–12] and, in our patient, at baseline, and it fell from 12 to 3.5 off treatment. We did not find increase in the ratio of urinary cortisol to cortisone metabolites in this or our previously reported patient, but it was present in one case reported by Thompson et al. [12].

Our previous report [11] concluded that inhibition of both enzymes is likely to be contributory to the mineralocorticoid excess and calculated that DOC increase due to 11*β*-hydroxylase inhibition could account for a significant proportion of the effect. Thompson et al. in a more recent publication of two new cases considered that inhibition of 11*β*-hydroxylase enzyme predominated in one patient on posaconazole treatment for coccidiomycosis and of 11*β*-HSD2 enzyme in the other patient on posaconazole prophylaxis for rhinocerebral mucormycosis [12]. In none did they find DOC increase present, in strong contrast to our findings and those of Barton et al. [9]. Our urine steroid profile findings closely resemble those we find in patients with the genetic disorder or on treatment with known 11*β*-hydroxylase inhibitors such as metyrapone and ketoconazole, so we believe that there is no reason to doubt that DOC increase would be an invariant feature. Ketoconazole has been reported to also inhibit cholesterol side chain cleavage and 17-hydroxylase, and the latter, by increasing flux in the mineralocorticoid relative to the glucocorticoid pathway, may account for a greater increase of DOC relative to 11-deoxycortisol, and thus, more severe hypertension than is usually associated with CAH due to 11*β*-hydroxylase deficiency.

It is not possible to determine the relative contributions to the effects of mineralocorticoid excess of these two induced enzyme deficiencies. Urine steroid profiling shows an obvious deficiency of 11*β*-hydroxylase and does not support a marked deficiency of 11*β*-HSD2, but, as discussed above, this does not preclude a more subtle but still clinically significant deficiency. Deficiency of 11*β*-HSD2 is associated with a greatly diminished cortisol clearance rate, which results in a diminished cortisol production rate and so would predict adrenocortical hypoplasia, whereas bilateral adrenal hyperplasia has been shown in animal studies of posaconazole action [19], supporting a predominant influence of the 11*β*-hydroxylase deficiency.

In the cases reported, the hypertension and hypokalaemia have been severe, requiring in some cases deployment of several antihypertensive agents, so it is surprising that these effects have received no mention in the report of the European Medicines Agency on 531 adult patients receiving doses of 800 mg/day [19]; the company's prescribing information quotes a rate of 11% hypertension and 22% hypokalaemia on a 300 mg dose [11]. Reported blood levels of posaconazole in the affected cases (all receiving 300 mg/day) vary from 3.0 to 4.9 mmol/L [9, 10, 12, 16], and the effects are dose-dependent [10, 12]. The sequence of events in some of the reported cases demonstrates that hypertension and hypokalaemia were only detected after other investigations had been completed. It therefore seems probable that this is an under-recognized problem.

Given the clinical utility of posaconazole, a practical solution to counter the hypertensinogenic effects may be to give spironolactone concomitantly, as we have previously suggested [11]. In the case of cystic fibrosis, the common use of systemic glucocorticoids may be unthinkingly achieving the same objective by preventing adrenocortical hyperplasia. If indeed the hypertensinogenic effect could be abolished only by glucocorticoids, this would favour the interpretation that 11*β*-hydroxylase inhibition is the primary cause of the mineralocorticoid excess; if it were 11*β*-HSD2 inhibition, the effect would be exacerbated.

High urinary catecholamine levels have been demonstrated in 11*β*-HSD2 knock-out mice models [20]. They were normal in our patient, in contrast to one reported finding [9]. The authors considered that this might be a consequence of concomitant treatment with nicardipine and esmolol, either by interference or by *β*-adrenergic stimulation of catecholamine secretion. As a potential contributor to the hypertension, this merits monitoring in further patients under treatment with posaconazole.

To conclude, evaluation of hypertension in our patient implicated posaconazole as the cause of excess mineralocorticoid action, predominantly due to inhibition of 11*β*-hydroxylase. The prevalence of hypertension in posaconazole-treated patients is unclear. As far as we are aware, this is the first report of posaconazole-induced hypertension in a young child with cystic fibrosis. We have reviewed the biochemical findings, in comparison with the limited number of previous reports, suggesting the possibility of a dose-dependent effect of posaconazole. There is a need for further research to explore the biological basis of interindividual variation in the nature and the severity of enzyme inhibition secondary to posaconazole treatment. We recommend an evaluation for cortisol insufficiency in patients who develop hypertension during posaconazole treatment.

## Figures and Tables

**Table 1:** Clinical and biochemical parameters on and off posaconazole treatment.

Parameters	On posaconazole	Off posaconazole	Normal reference range
Blood pressure (mm Hg)	150/84	92/56	95/56
Urine 11-deoxycorticosterone metabolite (*μ*g/mmol creatinine)	32	1.0	<1
Urine 11-deoxycortisol metabolite (*μ*g/mmol creatinine)	261	11.7	<15
Plasma renin (mU/L)	2.0	N/A	15.8–100.8
Plasma aldosterone (pmol/L)	45.0	N/A	80–970
Serum cortisol on SST^*∗*^ (nmol/L)	420^*∗∗*^	735^*∗∗*^	>500
Serum cortisone on SST^*∗*^ (nmol/L)	41^*∗∗*^	55^*∗∗*^	19.7–77.3
Cortisol : cortisone ratio	12^*∗∗*^	3.5^*∗∗*^	1.0–10.5
17-hydroxyprogesterone on SST^*∗*^ (nmol/L)	5^*∗∗*^	7.5^*∗∗*^	<30
Serum 11-deoxycortisol on SST^*∗*^ (nmol/L)	102^*∗∗*^	5^*∗∗*^	<2.7
Serum 11-deoxycorticosterone on SST (nmol/L)	41^*∗∗*^	1.6^*∗∗*^	<1.4
Serum corticosterone on SST^*∗*^ (nmol/L)	71^*∗∗*^	106^*∗∗*^	3.5–59.2
Posaconazole assay (mg/L)	6	N/A	1–3.75

^*∗*^Standard synacthen test; ^*∗∗*^peak concentration quoted. N/A: not available.

## Additional Points

*What is already known?* Posaconazole is known to cause both hypertension and hypokalaemia. The mechanism of posaconazole-induced hypertension is unclear, although reversible inhibition of 11*β*-hydroxylase and 11*β*-hydroxysteroid dehydrogenase type 2 enzymes has been proposed as the most likely mechanism. *What this study adds?* We report, for the first time, posaconazole-induced reversible hypertension and adrenal insufficiency in a young child with cystic fibrosis. Close monitoring for side effects, including blood pressure, electrolyte abnormalities and cortisol deficiency in children treated with long-term posaconazole therapy is essential.
